# Neuronal intranuclear inclusion disease presented with recurrent vestibular migraine-like attack: a case presentation

**DOI:** 10.1186/s12883-021-02367-6

**Published:** 2021-09-03

**Authors:** Danhua Zhao, Sha Zhu, Qinlan Xu, Jianwen Deng, Zhaoxia Wang, Xianzeng Liu

**Affiliations:** 1 Department of Neurology, Peking University International Hospital, 1 Shengmingyuan Road, Changping District, 102206 Beijing, P. R. China; 2 Department of Neurology, Peking University First Hospital, 100034 Beijing, China

**Keywords:** Neuronal intranuclear inclusion disease, Vestibular migraine, Episodic encephalopathy

## Abstract

**Background:**

Neuronal intranuclear inclusion disease (NIID) is a neurodegenerative disorder characterized by dementia, tremor, episodic encephalopathy and autonomic nervous dysfunction. To date, vestibular migraine (VM)-like attack has never been reported in cases with NIID. Here, we describe an 86-year-old patient with NIID who presented with recurrent vertigo associated with headache for more than 30 years.

**Case presentation:**

An 86-year-old Chinese woman with vertigo, headache, weakness of limbs, fever, and disturbance of consciousness was admitted to our hospital. She had suffered from recurrent vertigo associated with headache since her 50 s,followed by essential tremor and dementia. On this admission, brain magnetic resonance imaging revealed high intensity signals along the corticomedullary junction on diffusion weighted imaging (DWI). Peripheral neuropathy of the extremities was detected through electrophysiological studies. We diagnosed NIID after detecting eosinophilic intranuclear inclusions in the ductal epithelial cells of sweat glands and identifying an abnormal expansion of 81 GGC repeats in the 5’UTR of *NOTCH2NLC* gene.

**Conclusions:**

VM-like attack may be associated with NIID.

## Background

Vestibular migraine (VM) is an episodic disorder associated with both vestibular symptoms caused by migraine and the typical symptoms of migraine. It is considered as one of the most common causes of recurrent vertigo, with a lifetime prevalence about 1 % among the general population [1].

Neuronal intranuclear inclusion disease (NIID) is a clinically heterogeneous disorder mainly manifested with dementia, tremor, episodic encephalopathy, muscle weakness, and autonomic nervous dysfunction. NIID usually onsets at 50s or older in the sporadic cases and at 40s or younger in the familial cases [2]. Pathologically, it is characterized by eosinophilic ubiquitin-positive and p62-positive intranuclear inclusions in the neuronal and somatic cells of multiple systemic organs [2]. The distinct, high intensity signals along the corticomedullary junction on diffusion weighted imaging (DWI) of brain magnetic resonance imaging (MRI) has been shown to be a reliable, diagnostic marker of NIID [2]. Positive diagnoses of NIID have increased with the recognition of imaging features, the adoption of skin biopsy, and the discovery that the GGC repeated expansion in the 5’-untranslated region (5’UTR) of the *NOTCH2NLC* gene as a causative factor [3, 4]. However, VM-like attack has never been reported in cases with NIID. Here, we describe an 86-year-old patient with NIID who presented with recurrent vertigo associated with headache for more than 30 years.

## Case report

An 86-year-old Chinese woman admitted to our clinic, presenting with vertigo, headache, and vomiting for four days. She successively developed weakness in the left limbs, then the right limbs, fever, and disturbance of consciousness two days before admission. On examination, Glasgow coma scale on arrival was 7/15 (E2V1M4). She had normal pupils, reactive to light. Hypotonia and diffuse muscle atrophy in the limbs were observed. Deep tendon reflexes could not be elicited. There were no pyramidal signs except for a positive Chaddock’s sign on the left. Signs of meningeal irritation were negative.

Retrospectively, the patient had suffered from recurrent spontaneous vertigo since her 50 s. Initially, the symptom would be relieved following vomiting, lasting for half a day. With increasing age, the vertigo attacks were accompanied by severely pulsatile pain involving one or both sides of the head, photophobia, and phonophobia. The occurrence of attacks increased from once a year to 5–6 times a year, and the duration increased to 1–2 days. There were some triggers for the attacks, such as chocolates and sweet cakes. Initially, repeated brain CT scans showed no abnormalities, resulting in the recommendation of painkillers and anti-vertigo medicines to alleviate symptoms. In addition, in her 60 s, the patient gradually developed shaking of the hands when holding objects. By age 77, the patient noticed a deterioration in memory. Her family reported that she became increasingly stubborn and developed visual hallucinations. Several years ago, brain MRIs were performed, however, unfortunately, the results were unknown except for mild cerebral atrophy. She had no known family history of neurological diseases.

**Fig. 1:**
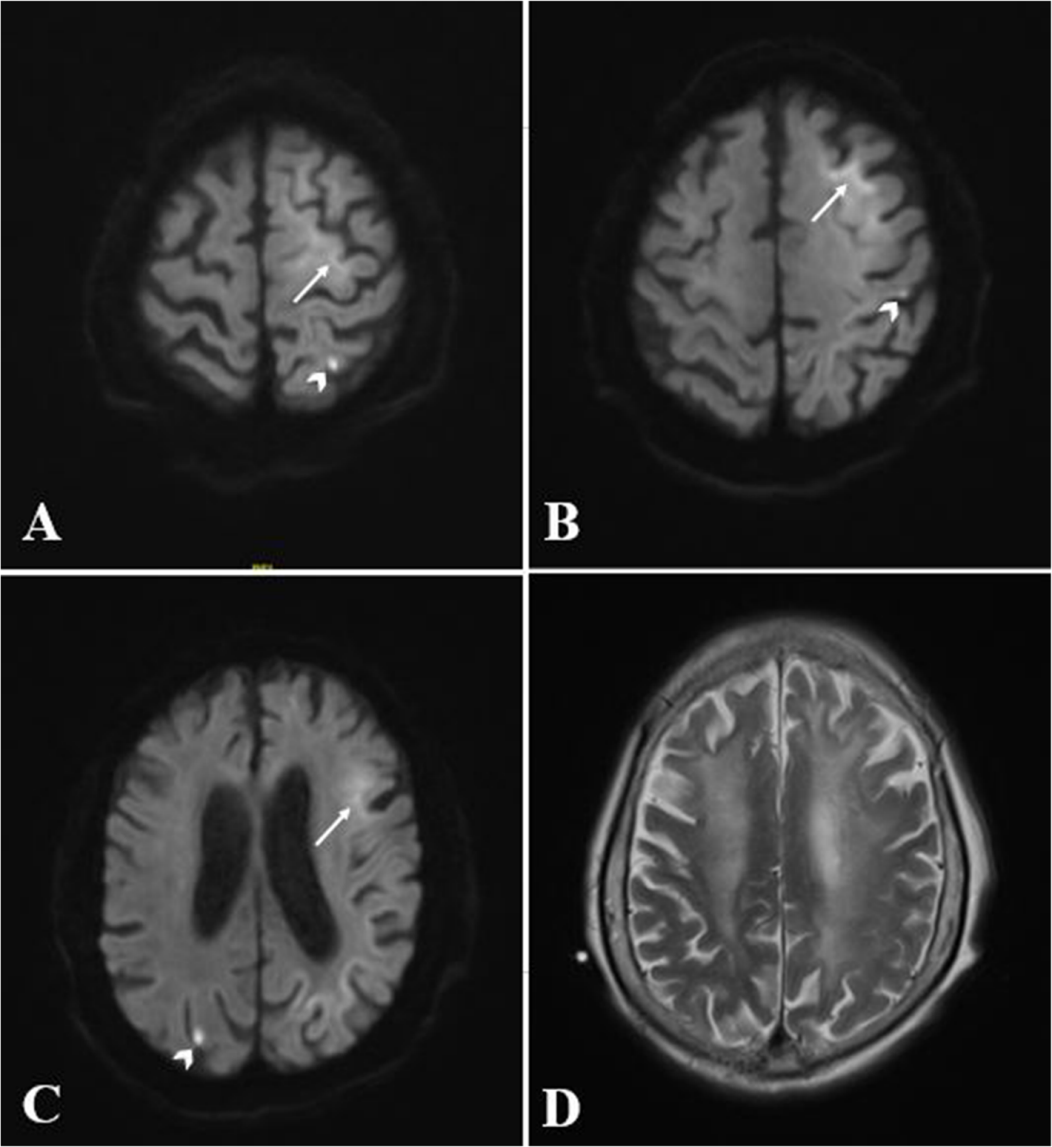
Brain MRI findings in an 86-year-old patient with NIID. Brain MRI reveals asymmetrical, curved, high signals along the corticomedullary junction on DWI (arrow, **A**, **B** and **C**), punctate high intensity signals in the cortex on DWI (arrowhead, **A**, **B** and **C**), and diffuse high signals of white matter on T2-weighted images (**D**)

Laboratory investigation showed a significant increase in leukocytes in both peripheral blood and urine, as well as elevated C-reactive protein, indicating a urinary tract infection. D-dimer was also substantially increased, up to 40,670 ng/mL (normal range < 250ng/ml), and pulmonary embolism (PE) of middle lobe in the right lung was confirmed by computed tomographic pulmonary angiography on the first day of admission. No deep venous thrombosis was detected in lower limbs by venous ultrasonography. Brain MRI revealed curve-like high intensity signals on DWI along the corticomedullary junction of the left hemisphere, particularly in the frontal lobe, as well as multifocal, punctate regions of high intensity signals on DWI and low signals on apparent diffusion coefficient (ADC) involving the bilateral cerebral cortex. Leukoencephalopathy and cerebral atrophy were also observed (Fig. 1). Color Doppler ultrasound revealed atherosclerotic plaques in carotid arteries and declined blood flow velocity in the intervertebral segment of right vertebral artery. Brain Magnetic Resonance Angiography (MRA) showed multiple mild to moderate stenosis of intracranial arteries, especially the right vertebral artery the most prominent. Digital video electroencephalogram revealed a small number of diffuse slow waves in all the leads. Nerve velocity conduction tests disclosed both axon and myelin damage in the nerves of the limbs.

**Fig. 2:**
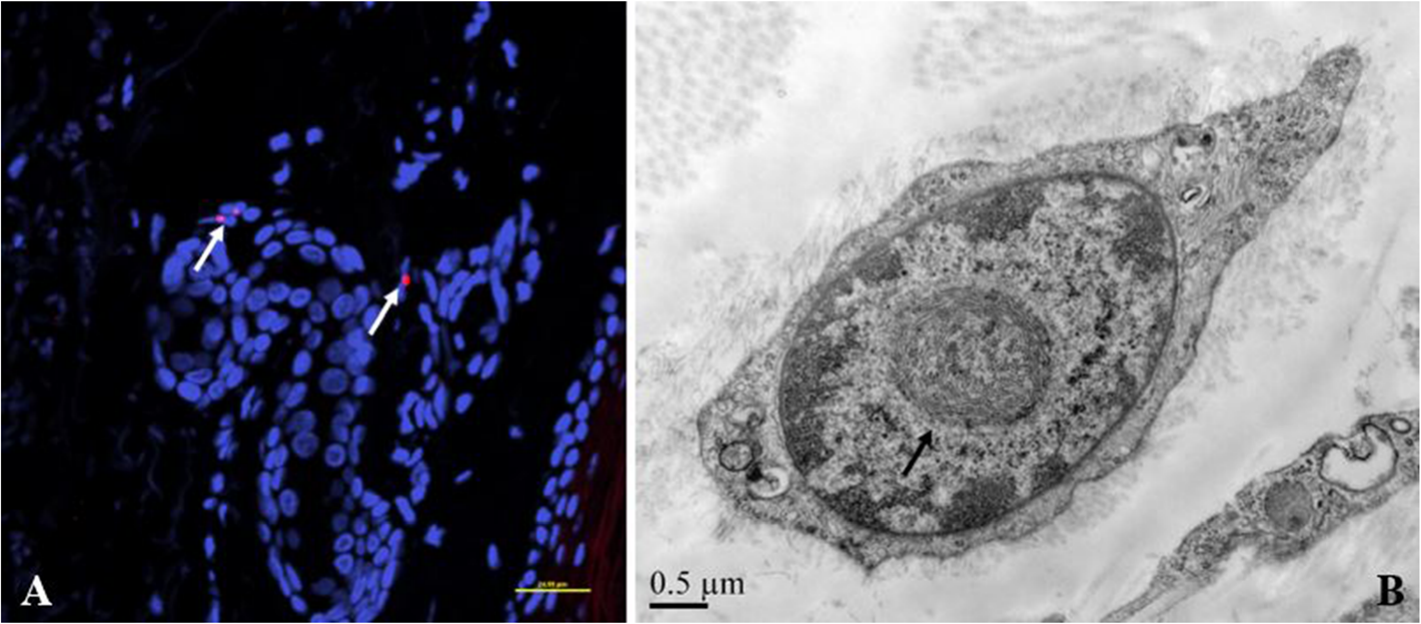
Pathological features in skin biopsy. Double immunofluorescence shows small red spheres indicating immunopositivity to p62 antibody in the nucleus (blue) (**A**, arrow). Electron microscopy shows the intranuclear inclusions are non-membrane bound filamentous materials in the center of the nucleus (**B**, arrow)

**Fig. 3:**
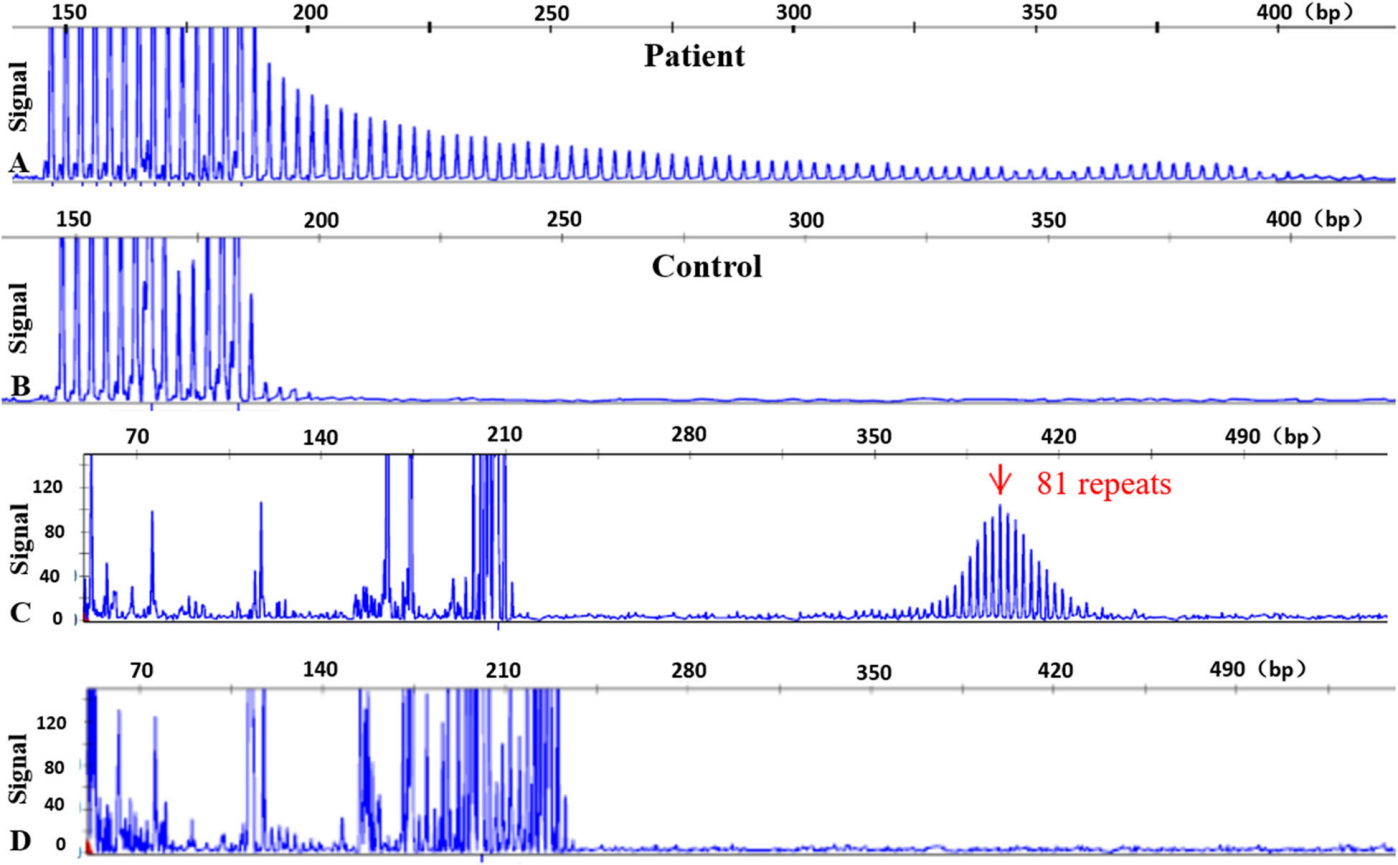
Genetic analysis of the *NOTCH2NLC* gene in the patient. On RP-PCR, the patient panel shows a saw-tooth tail pattern indicating an abnormal expansion more than 60 repeats (**A**), while control panel shows no repeat expansion in an unaffected individual (**B**). On fluorescence AL-PCR, expanded allele had an unusual peak at around 400 bp (**C**), while non-expanded allele had peak at around 240 bp (**D**)

Following the finding of high intensity signals on DWI along the corticomedullary junction, an imaging marker of NIID, skin biopsy and genetic analysis were performed, with written consent by the patient’s family. Skin biopsy revealed eosinophilic intranuclear inclusions in the ductal epithelial cells of sweat glands which were immunoreactive to anti-p62 antibodies. Electron microscopy disclosed filamentous material that lacked a limiting membrane in the nucleus (Fig. 2). Repeat-primed PCR (RP-PCR) and fluorescence amplicon length PCR (AL-PCR) were performed successively as described in previous study to identified 81 GGC repeats in the 5’-UTR [5, 6] (Fig. 3).

After admission, a urinary catheter was placed for treating dysuria and 1000 ml urine was drained after the catheterization, suggesting severe urinary retention. Antibiotics (cefoperazone 3 g, q12h for 7 days) and anticoagulant therapeutics (low molecular weight heparin 5000U, q12h for 7 days, followed by oral anticoagulation rivaroxaban 15 mg/d) were applied. The patient rapidly recovered to the clinical baseline by three days. When she was alert, neuropsychological tests were performed, for which she showed an MMSE score of 12 and a FAB score of 7. Cognitive impairment deteriorated accompanied by obvious psychiatric symptoms within one-year after discharge (from May 2020 to April 2021). During the same follow-up period, the patient complained of episodic vertigo, headache, and vomiting (4 times, lasting for 2–3 days per time), but no fever or neurological dysfunctions were noticed.

## Discussion

NIID is a heterogeneous disease and it is challenging to make an early diagnosis before episodic encephalopathy, an important diagnostic indicator, is identified. We described here a NIID case initially considered as ischemic brainstem infarction due to acute-onset vertigo, limb weakness and disturbance of consciousness. In this case, no lesions related to aforementioned symptoms in the brainstem were found on MRI. Instead, surprisingly, the unilateral, high intensity signals in the corticomedullary junction, another suggestive indicator of NIID, were observed on DWI. Combined with other typical clinical manifestations, including essential tremor, dementia, bladder dysfunction and peripheral neuropathy, NIID was considered. This diagnosis was ultimately confirmed by pathological and genetic analysis.

It should be noted that there are some atypical clinical presentations in the patient. First of all, it is intriguing that the patient developed recurrent vertigo associated with headaches since her 50 s. Based on the clinical features of vertigo and headache, probable VM was considered in accordance with the diagnostic criteria proposed by the joint committee of the International Headache Society and Barany Society [7]. Although vestibular testing was absent in this case, vestibular symptoms associated with migraine features provided the key to diagnosis.

As far as we know, VM has never been described in the patients with NIID, although other subtypes of migraine have been reported previously. Wang described a juvenile NIID patient who developed probable migraine without aura, migraine with aura, and hemiplegic migraine before episodic encephalopathy [8]. Liang reported a 35-year-old female patient with NIID who presented with migraine with aura as an initial symptom [6]. These cases raise a question whether there is any linkage between the two disorders or just a coincidence. The evolution of clinical symptoms in these cases, from the earlier isolated migraine-like attack to the later migraine-like attack associated with disturbance of consciousness in the episodic encephalopathy, suggest a coherent process of one disease at different stages. However, the initiating factor of the disease remains unclear. Wang et al. have proposed two possibilities based on the their clinical data: the first is that cerebral hypoperfusion in HM leading to the depletion of oxygen and nutrients in the cerebrum which may contribute to the accumulation of eosinophilic inclusions, and subsequently resulting in progression of NIID; the second is that the accumulation of eosinophilic inclusions happen first and lead to the development of vascular dysfunction, resulting in HM attacks [8]. In this case, it is hard to draw either conclusion due to lack of assessment of cerebral blood flow perfusion. Additionally, because VM is a common clinical disease, the possibility that VM and NIID exist as 2 independent clinical conditions cannot be fully ruled out. More cases and further research are needed to explore the relationship between the two disorders.

Secondly, it is interesting that the episodic encephalopathy coexisted with both arterial and venous thrombosis. On the one hand, multifocal punctate cortical lesions with high signals on DWI and low signals on ADC were found on brain MRI. Based on the radiological features, acute cerebral infarctions in the posterior circulation were considered, which may be due to embolization from the right vertebral artery. In addition, hypercoagulability was considered in this case because of the significantly elevated plasma D-dimer, the possibility of hypercoagulability resulting in ischemic stroke thus could not be entirely excluded. However, these small infarctions could not explain the unconsciousness and limb weakness in the case. On the other hand, the case developed PE which was also attributed to hypercoagulability. The clinical presentations of PE are highly variable, including syncope caused by obstructive shock. However, the case appeared hemodynamically stable during disturbance of consciousness, which is difficulty to attribute to PE. Therefore, it is more reasonable to explain the stroke-like attack with episodic encephalopathy of NIID rather than thrombotic diseases.

Based on the available literature, no definite association with hypercoagulability was found in cases with NIID, however, a growing body of evidence suggested that migraine, especially migraine aura, linked to hypercoagulability. Several mechanisms underlying the linkage were proposed, including ischemia-induced cortical spread depression, weakening of the blood brain barrier, endothelial damage, inflammation and stress [9]. It is uncertain whether there was a relationship between vestibular migraine and hypercoagulability in our case. Furthermore, the possibility that hypercoagulability developed during prolonged bed rest (4 days before admission) due to episodic encephalopathy could not be excluded.

The major limitation of this case is the absence of brain MRI scans at different disease stages, thus it is difficult to establish a correlation between clinical and radiological features or to understand how the disease evolved.

## Conclusions

In conclusion, VM-like attack may be associated with NIID. For elderly patients with new-onset migraine-like attack, especially accompanied by other neurodegenerative symptoms, NIID should be considered.

## Data Availability

The datasets used and/or analyzed during the current study are available from the corresponding author on reasonable request.

## Abbreviations

NIID: Neuronal intranuclear inclusion disease;
VM: Vestibular migraine
DWI: Diffusion weighted imaging
ADC: Apparent diffusion coefficient
MRI: Magnetic resonance imaging
5’UTR: 5’-untranslated region
RP-PCR: Repeat-primed Polymerase Chain Reaction
AL-PCR: amplicon length Polymerase Chain Reaction
